# Histogram analysis of multiple diffusion models for predicting advanced non-small cell lung cancer response to chemoimmunotherapy

**DOI:** 10.1186/s40644-024-00713-8

**Published:** 2024-06-11

**Authors:** Yu Zheng, Liang Zhou, Wenjing Huang, Na Han, Jing Zhang

**Affiliations:** 1 Department of Magnetic Resonance, The Second Hospital & Clinical Medical School, Lanzhou University, Lanzhou, 730030 China; 2 Gansu Province Clinical Research Center for Functional and Molecular Imaging, Lanzhou, 730030 China

**Keywords:** Intravoxel incoherent motion, Diffusion kurtosis imaging, Histogram analysis, Non-small cell lung cancer, Chemoimmunotherapy

## Abstract

**Background:**

There is an urgent need to find a reliable and effective imaging method to evaluate the therapeutic efficacy of immunochemotherapy in advanced non-small cell lung cancer (NSCLC). This study aimed to investigate the capability of intravoxel incoherent motion (IVIM) and diffusion kurtosis imaging (DKI) histogram analysis based on different region of interest (ROI) selection methods for predicting treatment response to chemoimmunotherapy in advanced NSCLC.

**Methods:**

Seventy-two stage III or IV NSCLC patients who received chemoimmunotherapy were enrolled in this study. IVIM and DKI were performed before treatment. The patients were classified as responders group and non-responders group according to the Response Evaluation Criteria in Solid Tumors 1.1. The histogram parameters of ADC, Dslow, Dfast, f, Dk and K were measured using whole tumor volume ROI and single slice ROI analysis methods. Variables with statistical differences would be included in stepwise logistic regression analysis to determine independent parameters, by which the combined model was also established. And the receiver operating characteristic curve (ROC) were used to evaluate the prediction performance of histogram parameters and the combined model.

**Results:**

ADC, Dslow, Dk histogram metrics were significantly lower in the responders group than in the non-responders group, while the histogram parameters of f were significantly higher in the responders group than in the non-responders group (all *P* < 0.05). The mean value of each parameter was better than or equivalent to other histogram metrics, where the mean value of f obtained from whole tumor and single slice both had the highest AUC (AUC = 0.886 and 0.812, respectively) compared to other single parameters. The combined model improved the diagnostic efficiency with an AUC of 0.968 (whole tumor) and 0.893 (single slice), respectively.

**Conclusions:**

Whole tumor volume ROI demonstrated better diagnostic ability than single slice ROI analysis, which indicated whole tumor histogram analysis of IVIM and DKI hold greater potential than single slice ROI analysis to be a promising tool of predicting therapeutic response to chemoimmunotherapy in advanced NSCLC at initial state.

**Supplementary Information:**

The online version contains supplementary material available at 10.1186/s40644-024-00713-8.

## Background

Lung cancer is leading cause of cancer-related deaths worldwide, and the 5-year overall survival rate is approximately 20% [1]. Non-small cell lung cancer (NSCLC) accounts for approximately 85% of lung cancers [1], and most NSCLC is discovered at an advanced stage. Immune-checkpoint inhibitors (ICIs) improve the outcomes of NSCLC patients, and a combination of immunotherapy and chemotherapy increases the efficacy over chemotherapy alone [2, 3]. Programmed death-ligand 1 (PD-L1) protein expression has been considered as a predictive biomarker for immunotherapy in NSCLC patients [4]. However, PD-L1 expression cannot fully predict benefit of therapy, and some patients can respond even with low or negative PD-L1 expression, particularly for those treated with immunotherapy-based combinations [5]. Thus, there is a pressing need to find new biomarkers to predict tumor response to chemotherapy combined with immunotherapy.

Diffusion-weighted imaging (DWI) is an effective means of reflecting tissue water molecular restriction, and has been widely used to assess treatment outcomes in lung cancer patients [6–9]. However, conventional monoexponential DWI does not give good account of non-Gaussian diffusion [10], and is influenced by microcirculation, thus does not accurately reflect true water diffusion [11]. The intravoxel incoherent motion (IVIM) technique proposed by Le Bihan et al. [12] can independently assess the diffusion of water molecules and tissue microcirculation. Several studies have demonstrated that IVIM has been used to evaluate the efficacy of treatment for lung cancer [13–15]. Moreover, diffusion kurtosis imaging (DKI) is a model that reflects the non-Gaussian distribution of water molecules and the complexity of tissue microstructures [16]. DKI is currently used to differentiate lung cancer lesions, and evaluate EGFR mutations and histopathological features of lung cancer [11, 17, 18]. To our knowledge, there is currently only one study using DKI to predict the therapeutic efficacy of lung cancer [19] .

Furthermore, most previous studies measured parameters on a representative section of tumor tends to underestimate the heterogeneity of the tumor. Histogram analysis of the whole tumor is able to reflect the distribution and variation of all voxels within the whole lesion, and detect the heterogeneity of tumors, thereby eliminating sampling bias and providing reproducible results [20]. In order to save time, single slice histogram analysis was often used. One study [21] found that whole-tumor volume is preferred over single-section region of interest (ROI) analysis when evaluating the treatment outcomes of rectal cancer. To our knowledge, no published studies using histogram analysis of IVIM and DKI for early prediction of tumor response to chemoimmunotherapy in NSCLC, and there is also a lack of comparison between different measurement methods.

Thus, the aim of our study was to investigate the capability of conventional DWI, IVIM and DKI histogram parameters obtained by using whole tumor volume ROI and single slice ROI methods for predicting treatment response to chemoimmunotherapy in advanced NSCLC.

## Methods

### Patients

This prospective study was approved by the Ethics Review Board of our hospital, and written informed consent was obtained from each patient. Between December 2021 and June 2023, 83 consecutive patients pathologically diagnosed with advanced NSCLC underwent pretreatment MRI examination with IVIM and DKI sequences. The inclusion criteria were as follows: (1) histological diagnosis of NSCLC; (2) stage III or IV based on TNM staging system of American Joint Committee on Cancer (AJCC) 8th ; (3) received chemoimmunotherapy; (4) Eastern Cooperative Oncology Group (ECOG) score of 0 to 1; (5) Without any anti-tumor treatment. The exclusion criteria were the following: (1) MRI contraindications; (2) incompleted chemoimmunotherapy; (3) inferiorquality of IVIM or DKI images. Finally, 72 patients were enrolled in this study.

### MRI acquisition

All patients underwent MR scanning within 1 week before biopsy and treatment. All the examinations were performed on a 3.0-T system (GE Signa Premier 3.0T MRI scanner, GE Healthcare, USA) using respiratory gating to reduce motion artifacts. Routine MRI sequences, IVIM and DKI were performed in sequence. Routine MRI sequences include coronal single shot fast spin-echo T2-weighted (T2W) image (repetition time/echo time [TR/TE], 2608/80 ms; slice thickness, 6 mm; spacing, 1 mm; field of view [FOV], 400 × 400 mm; matrix, 320 × 256), transverse respiratory-triggered T2W with fat suppression (TR/TE, 10,000/85 ms; slice thickness, 5 mm; spacing, 1 mm; FOV, 380 × 380 mm; matrix, 288 × 288), and axial T1-weighted (T1W) breath-hold liver acquisition with volume acceleration (LAVA) image (TR/TE, 2.60/1.14 ms; slice thickness, 1.4 mm; FOV, 380 × 380 mm; matrix, 272 × 224).

Axial IVIM was performed with a single-shot echo-planar imaging pulse sequence (TR/TE, 7500/63 ms; slice thickness, 4 mm; spacing, 1 mm; FOV, 380 × 380 mm; matrix, 256 × 256; bandwidth, 250 kHz/pix; ASSET = 2; b values, 0, 20, 50, 80, 150, 200, 400, 600, 800,and 1000 s/mm^2^). The acquisition time depended on the respiratory rhythm of the patient, ranging from 5 to 8 min. Axial DKI was acquired by using three b values that ranged from 0 to 2000 s/mm^2^ (0, 1000, 2000 s/mm^2^) with the following parameters: TR/TE, 6923/65 ms; slice thickness, 4 mm; spacing, 1 mm; FOV, 380 × 380 mm; matrix, 256 × 256; bandwidth, 250 kHz/pix.

### Image postprocessing and analysis

The apparent diffusion coefficient (ADC) was obtained by using a monoexponential model of DWI with the following equation [22] :

$${\rm{S(b)/S(0)}}\,{\rm{ = }}\,{\rm{exp( - b}}\, \cdot \,{\rm{ADC),}}$$

The IVIM parameters including the true diffusion coefficient (Dslow), the pseudo-diffusion coefficient (Dfast), and the perfusion fraction (f) were calculated with the following [12] :

$$S(b)/S(0) = [(1 - f) \cdot \exp ( - b \cdot Dslow)] + [f \cdot exp( - b \cdot (Dslow + Dfast))]$$

In the DKI model, the parameters including the corrected diffusion coefficient (Dk) and the diffusion kurtosis value (K) were derived using the following equation [16] :

$${\rm{S(b)/S(0)}}\,{\rm{ = }}\,{\rm{exp( - b}}\, \cdot \,{\rm{Dk}}\,{\rm{ + }}\,{{\rm{b}}^2}\, \cdot \,{\rm{D}}{{\rm{k}}^2}\, \cdot \,{\rm{K/6),}}$$

where S(b) is the signal intensity in the b value and S(0) represents the signal intensity without diffusion gradient. All the original DWI data were post-processed using an in-house software (FireVoxel, https://firevoxel.org/).

Two methods (whole tumor and single slice) of ROI were used to measure DWI parameters. The largest tumor was selected for measurement. For whole tumor volume ROI analysis, Two radiologists (7 and 10 years of experience in throax imaging, respectively) who were blinded to the pathological results independently drew the ROI along the outer edge of the tumor solid components section by section on DWI (b = 0 s/mm^2^) to obtain a three-dimensional ROI. For single slice ROI analysis, the same two radiologists independently drew ROI to include tumor solid part on maximum cross-sectional slice. Necrosis, visible vessels, and hemorrhage were avoided with reference to T1WI and T2WI. Histogram parameters of ADC, Dslow, Dfast, f, Dk and K maps were automatically extracted from the whole tumor volume and single slice, including the mean, median, 10th, 25th, 75th, and 90th percentile values, skewness and kurtosis. The mean values of the two measurements were used for further quantitative statistical analysis.

### Response evaluation

Treatment response was evaluated on the basis of the Response Evaluation Criteria in Solid Tumors (RECIST Version1.1) [23]. The criteria for judging the therapeutic effect are as follows: complete response (CR), disappearance of all targeted lesions; partial response (PR), the total diameters of target lesions decreased by at least 30%; progressive disease (PD), the total diameters of target lesions increased by at least 20%; and stable disease (SD), neither sufficient shrinkage to qualify for PR nor sufficient increase to qualify for PD. All the patients received immunotherapy (tislelizumab or sintilimab or serplulimab) combined with platinum-based chemotherapy for 4 cycles, with one cycle lasting 21 days. Twelve weeks after the end of chemoimmunotherapy, the patients were classified as responders group (CR and PR) and non-responders group (PD and SD) according to RECIST Version1.1.

### Statistical analysis

Statistical analyses were performed using SPSS 22.0 (IBM SPSS Statistics, USA) and MedCalc 19.0.4 (MedCalc, Ostend, Belgium). Interobserver agreement was evaluated by intraclass correlation coefficient (ICC). The criteria are as follows: 0.00-0.20, poor correlation; 0.21–0.40, fair correlation; 0.41–0.60, moderate correlation; 0.61–0.80, good correlation; and 0.81-1.00, excellent correlation [21]. The Shapiro-Wilk test was used to evaluate the normality of data distribution. Unpaired Student’s t-test or Mann-Whitney U test were performed to evaluate the differences in continuous variables, as appropriate. Categorical variables were compared using chi-squared test. Variables with statistical differences will be included in stepwise logistic regression analysis to determine independent parameters, by which the combined model was also established. Receiver operating characteristic curve (ROC) analyses were used to evaluate the diagnostic performances of significant parameters and models. The area under the curve (AUC) were calculated and compared by using the method of DeLong. Moreover, cutoff value, sensitivity, specificity, accuracy, positive prediction value (PPV) and negative prediction value (NPV) were also computed. A P value less than 0.05 was considered statistical significance.

## Results

### Patients and tumors characteristics

The characteristics of patients and tumors are described in Table 1. A totally 72 patients (56 males and 16 females) were enrolled in this study, including 41 responders and 31 non-responders, mean age was 60.08 ± 8.67 years (range from 30 to 79). Adenocarcinoma was the most common histological type in this study (*n* = 30, 41.67%). The average size of the largest dimension of the tumors was 5.62 ± 1.88 cm. The most patients were in stage IIIB (*n* = 25, 34.72%), followed by stage IIIA and IIIC (both *n* = 15, 20.83%), stage IVA (*n* = 10, 13.89%), and stage IVB (*n* = 7, 9.72%). Most tumors had a low degree of differentiation (*n* = 45, 62.50%). The mean Ki67 index is 56.81 ± 26.21%, and the Ki67 index of the responders group was significantly higher than that of the non-responders group (63.66 ± 25.62% and 47.74 ± 24.52%, respectively) (*p* = 0.007). There were no statistical differences in age, sex, smoking history, histology, tumor size, clinical stage, differentiation degree and PD-1 inhibitors between the two grou*p*s (all *P* > 0.05).

**Table 1:** Patients and tumors characteristics

Characteristics	All	Responders (n = 41)	Non-responders (n = 31)	P
Age (year)	60.08 ± 8.67	58.78 ± 8.99	61.81 ± 8.04	0.144
Sex, n (%)				0.098
Female	16 (22.22%)	12 (29.27%)	4 (12.90%)	
Male	56 (77.78%)	29 (70.73%)	27 (87.10%)	
Smoking history, n (%)				0.463
Yes	29 (40.28%)	15 (36.59%)	14 (45.16%)	
No	43 (59.72%)	26 (63.41%)	17 (54.84%)	
Histology, n (%)				
Adenocarcinoma	30 (41.67%)	16 (39.02%)	14 (45.16%)	0.352
Squamous cell carcinoma	27 (37.50%)	14 (34.15%)	13 (41.94%)	
Other	15 (20.83%)	11 (26.83%)	4 (12.90%)	
Tumor size, cm	5.62 ± 1.88	5.66 ± 1.59	5.56 ± 2.24	0.826
Clinical stage, n (%)				0.810
IIIA	15 (20.83%)	7 (17.07%)	8 (25.81%)	
IIIB	25 (34.72%)	15 (36.59%)	10 (32.26%)	
IIIC	15 (20.83%)	9 (21.95%)	6 (19.35%)	
IVA	10 (13.89%)	5 (12.20%)	5 (16.13%)	
IVB	7 (9.72%)	5 (12.20%)	2 (6.45%)	
Ki67 (%)	56.81 ± 26.21	63.66 ± 25.62	47.74 ± 24.52	0.007
Differentiation degree, n (%)				0.243
Low	45 (62.50%)	28 (68.29%)	17 (54.84%)	
Moderately and highly	27 (37.50%)	13 (31.71%)	14 (45.16%)	
PD-1 inhibitors, n (%)				0.732
Tislelizumab	22 (30.56%)	11 (26.83%)	11 (35.48%)	
Sintilimab	30 (41.67%)	18 (43.90%)	12 (38.71%)	
Serplulimab	20 (27.78%)	12 (29.27%)	8 (25.81%)	

### Interobserver agreement evaluation

Given that the results of this study showed that the histogram parameters of Dfast and K were not helpful in predicting the efficacy of immunochemotherapy in advanced NSCLC, only the interobserver agreement of the histogram parameters of ADC, Dk, Dslow and f was evaluated. Those parameters showed good to excellent interobserver agreements in two different measurement methods, with ICC values range from 0.805 to 0.963. The detailed interobserver agreements for each histogram parameter are shown in Table 2.

**Table 2:** Interobserver agreement (ICC) for each parameter measurement

Histogram parameter	ICC (95% CI)	
	Whole tumor	Single slice
ADC		
10th	0.867 (0.795–0.914)	0.843 (0.761–0.899)
25th	0.851 (0.772–0.904)	0.837 (0.751–0.895)
75th	0.922 (0.879–0.951)	0.832 (0.744–0.891)
90th	0.832 (0.745–0.892)	0.823 (0.731–0.885)
Mean	0.918 (0.872–0.948)	0.916 (0.869–0.946)
Median	0.887 (0.825–0.928)	0.872(0.803–0.918)
Dslow		
10th	0.810 (0.713–0.877)	0.916 (0.869–0.946)
25th	0.906 (0.853–0.940)	0.843 (0.760–0.899)
75th	0.893 (0.835–0.932)	0.902 (0.848–0.938)
90th	0.916 (0.870–0.947)	0.871 (0.802–0.917)
Mean	0.911 (0.861–0.943)	0.844 (0.762-0.900)
Median	0.931 (0.892–0.956)	0.901 (0.846–0.937)
Dk		
10th	0.903 (0.849–0.938)	0.846 (0.765–0.901)
25th	0.923 (0.880–0.951)	0.881 (0.816–0.924)
75th	0.858 (0.782–0.909)	0.873 (0.804–0.919)
90th	0.805 (0.705–0.873)	0.891 (0.831–0.930)
Mean	0.807 (0.708–0.875)	0.831 (0.743–0.891)
Median	0.877 (0.811–0.921)	0.867 (0.795–0.914)
f		
10th	0.904 (0.851–0.939)	0.903 (0.850–0.938)
25th	0.918 (0.872–0.948)	0.902 (0.848–0.938)
75th	0.854 (0.776–0.906)	0.879 (0.813–0.922)
90th	0.830 (0.741–0.890)	0.963 (0.941–0.976)
Mean	0.902 (0.848–0.938)	0.921 (0.877–0.950)
Median	0.811 (0.714–0.877)	0.896 (0.839–0.934)

CI: confidence interval

### Comparisons of whole tumor ADC, IVIM and DKI histogram metrics

**Fig. 1:**
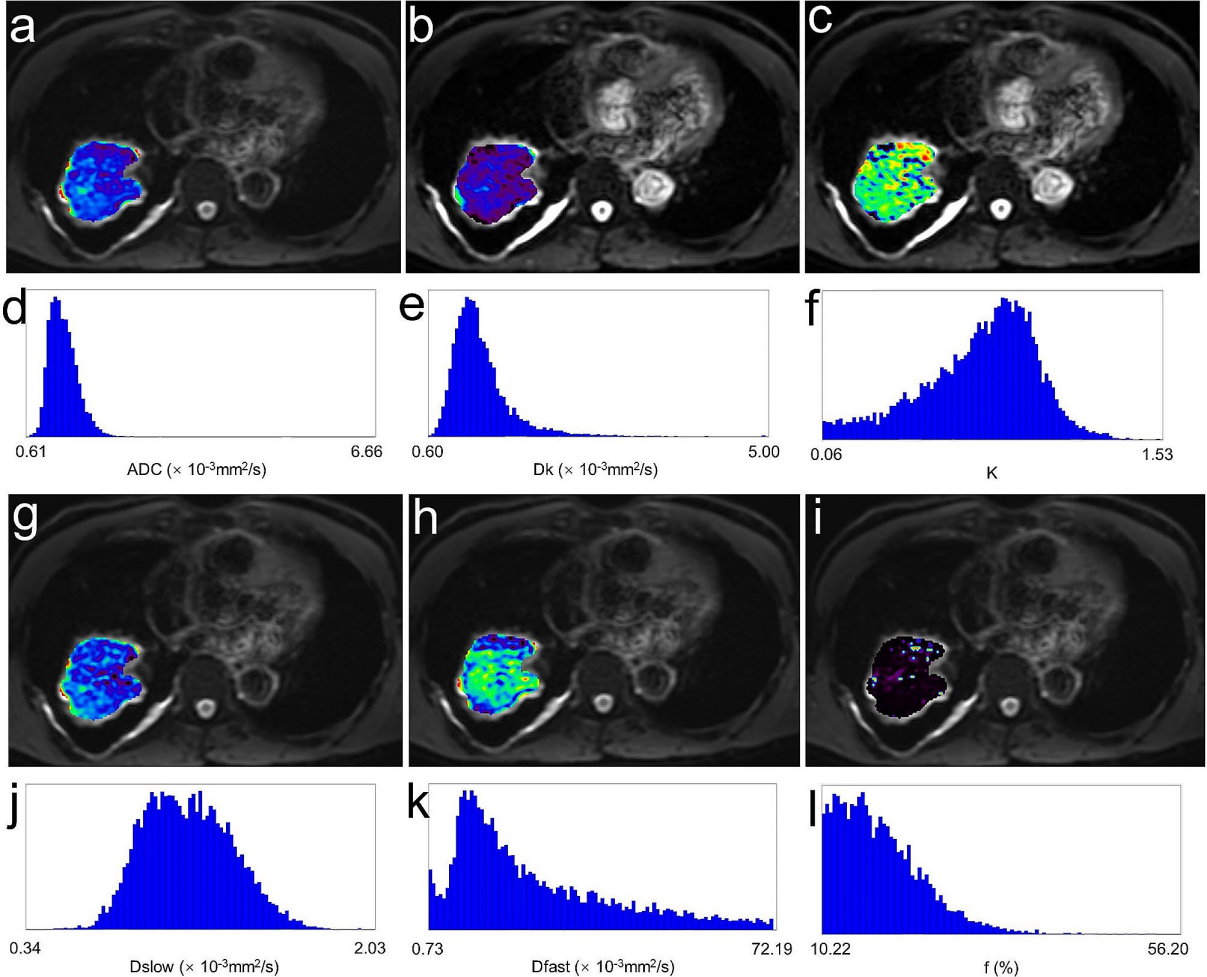
A 60-year-old male with lung adenocarcinoma with partial response (PR). Pre-treatment apparent diffusion coefficient (ADC) (**a**), corrected diffusion coefficient (Dk) (**b**), diffusion kurtosis value (K) (**c**), true diffusion coefficient (Dslow) (**g**), pseudo-diffusion coefficient (Dfast) (**h**), and perfusion fraction (**f**) (**i**) maps and their corresponding histograms (**d-f, j-l**) were obtained by whole tumor volume method

**Fig. 2:**
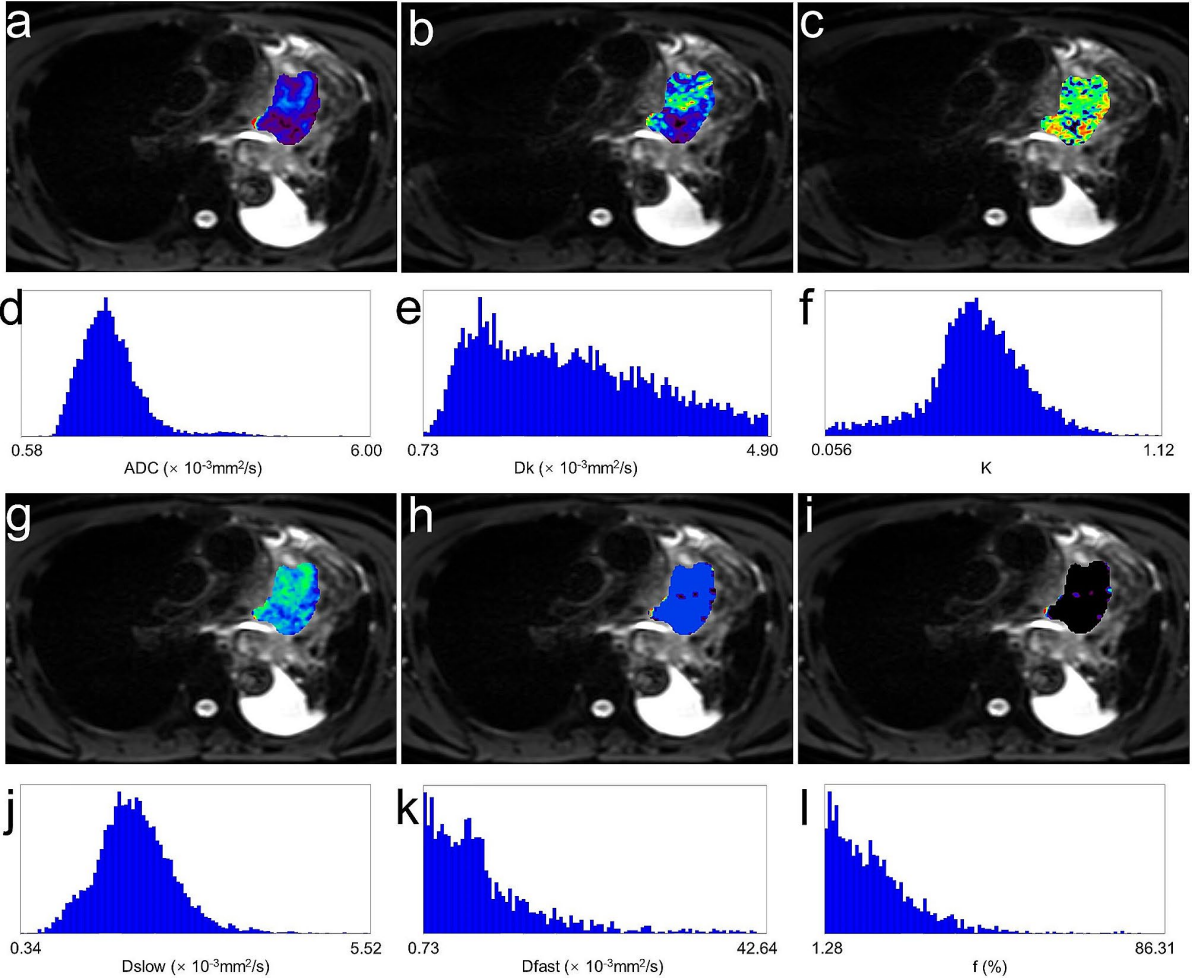
A 55-year-old male with lung squamous cell carcinoma with stable disease (SD). Pre-treatment apparent diffusion coefficient (ADC) (**a**), corrected diffusion coefficient (Dk) (**b**), diffusion kurtosis value (K) (**c**), true diffusion coefficient (Dslow) (**g**), pseudo-diffusion coefficient (Dfast) (**h**), and perfusion fraction (f) (**i**) maps and their corresponding histograms (**d-f, j-l**) were obtained by whole tumor volume method

A comparison of the ADC, IVIM and DKI histogram parameters obtained by whole tumor analysis between the responders and non-responders groups is shown in Tables 3 and 4. The histogram parameters of ADC (mean, median, 75th), Dk (mean, 75th, 90th), and Dslow (mean, median, 10th, 25th, 75th, 90th) in the responders group were significantly lower than those in the non-responders group, while the histogram metrics of f (mean, median, 25th, 75th, 90th) were significantly higher in the responders group than those in the non-responders group (all *P* < 0.05). In terms of the Dfast and K values, none of the histogram parameters differed significantly (all *P* > 0.05). Representative cases are shown in Figs. 1 and 2.

**Table 3:** Comparisons of ADC and DKI histogram metrics obtained by using whole tumor analysis

Parameters	ADC		P	Dk		P	K		P
	Responders (n = 41)	Non-responders (n = 31)		Responders (n = 41)	Non-responders (n = 31)		Responders (n = 41)	Non-responders (n = 31)	
10th	0.92 ± 0.12	0.96 ± 0.16	0.258	0.89 ± 0.15	0.93 ± 0.11	0.271	0.46 ± 0.14	0.42 ± 0.13	0.219
25th	1.12 ± 0.15	1.16 ± 0.20	0.299	1.11 ± 0.17	1.16 ± 0.14	0.195	0.72 ± 0.18	0.67 ± 0.18	0.200
75th	1.62 ± 0.22	1.75 ± 0.25	0.017	1.69 ± 0.18	1.77 ± 0.15	0.039	1.25 ± 0.29	1.14 ± 0.32	0.169
90th	1.89 ± 0.18	1.99 ± 0.24	0.053	1.93 ± 0.19	2.02 ± 0.15	0.038	1.45 ± 0.35	1.38 ± 0.41	0.446
Mean	1.40 ± 0.19	1.57 ± 0.25	0.001	1.45 ± 0.15	1.52 ± 0.13	0.032	0.98 ± 0.22	0.91 ± 0.24	0.216
Median	1.35 ± 0.22	1.47 ± 0.25	0.025	1.42 ± 0.17	1.49 ± 0.17	0.064	1.00 ± 0.24	0.91 ± 0.25	0.124
Skewness	1.55 ± 1.50	1.98 ± 2.14	0.258	0.41 ± 0.83	0.56 ± 0.96	0.484	-0.09 ± 0.68	0.24 ± 0.86	0.063
kurtosis	1.95 ± 2.23	3.01 ± 2.76	0.071	-0.05 ± 1.07	0.11 ± 1.26	0.838	0.51 ± 2.51	1.08 ± 2.10	0.055
Entropy	2.07 ± 1.06	2.30 ± 1.11	0.178	3.45 ± 0.23	3.54 ± 0.27	0.142	4.00 ± 0.25	3.92 ± 0.28	0.213

**Table 4:** Comparisons of IVIM histogram metrics obtained by using whole tumor analysis

Parameters	Dslow		*P*	Dfast			*P*	f		*P*
	Responders (n = 41)	Non-responders (n = 31)		Responders (n = 41)	Non-responders (n = 31)			Responders (n = 41)	Non-responders (n = 31)	
10th	0.77 ± 0.11	0.85 ± 0.10	0.004	3.10 ± 1.70	2.55 ± 1.07		0.303	4.29 ± 3.05	3.23 ± 2.02	0.082
25th	0.99 ± 0.12	1.07 ± 0.15	0.021	5.70 ± 2.24	4.93 ± 1.30		0.069	11.11 ± 5.09	7.06 ± 2.75	0.000
75th	1.46 ± 0.22	1.66 ± 0.24	0.000	15.64 ± 5.29	14.36 ± 3.90		0.261	34.41 ± 11.40	20.73 ± 7.29	0.000
90th	1.75 ± 0.25	1.94 ± 0.29	0.005	25.39 ± 7.98	23.77 ± 6.34		0.357	45.66 ± 12.19	32.70 ± 11.34	0.000
Mean	1.21 ± 0.19	1.45 ± 0.41	0.000	12.32 ± 3.78	11.07 ± 2.78		0.127	26.07 ± 9.51	14.25 ± 4.93	0.000
Median	1.20 ± 0.19	1.41 ± 0.21	0.000	9.61 ± 3.35	8.47 ± 2.12		0.081	22.80 ± 9.67	12.62 ± 4.80	0.000
Skewness	0.99 ± 1.05	1.22 ± 0.80	0.324	1.76 ± 0.85	1.76 ± 0.73		0.547	0.89 ± 0.67	1.15 ± 0.53	0.087
kurtosis	2.10 ± 2.62	2.42 ± 2.69	0.838	3.36 ± 4.05	3.70 ± 4.51		0.735	1.12 ± 1.94	1.31 ± 1.08	0.083
Entropy	3.49 ± 0.43	3.28 ± 0.59	0.076	1.39 ± 0.97	1.49 ± 1.18		0.692	3.93 ± 0.40	3.80 ± 0.30	0.115

### Comparisons of single slice ADC, IVIM and DKI histogram metrics

Based on the results of whole tumor volumn histogram analysis, single slice ROI analysis was conducted on the histogram parameters of ADC, Dk, Dslow and f. The histogram parameters of ADC (mean, median, 75th), Dk (90th), and Dslow (mean, median, 10th, 25th, 75th) in the responders group were significantly lower than those in the non-responders group, while the histogram metrics of f (mean, median, 25th, 75th, 90th) were significantly higher in the responders group than those in the non-responders group (all *P* < 0.05) (Supplementary Tables 1 and 2).

### The diagnostic performance of the two ROI selection methods

**Fig. 3:**
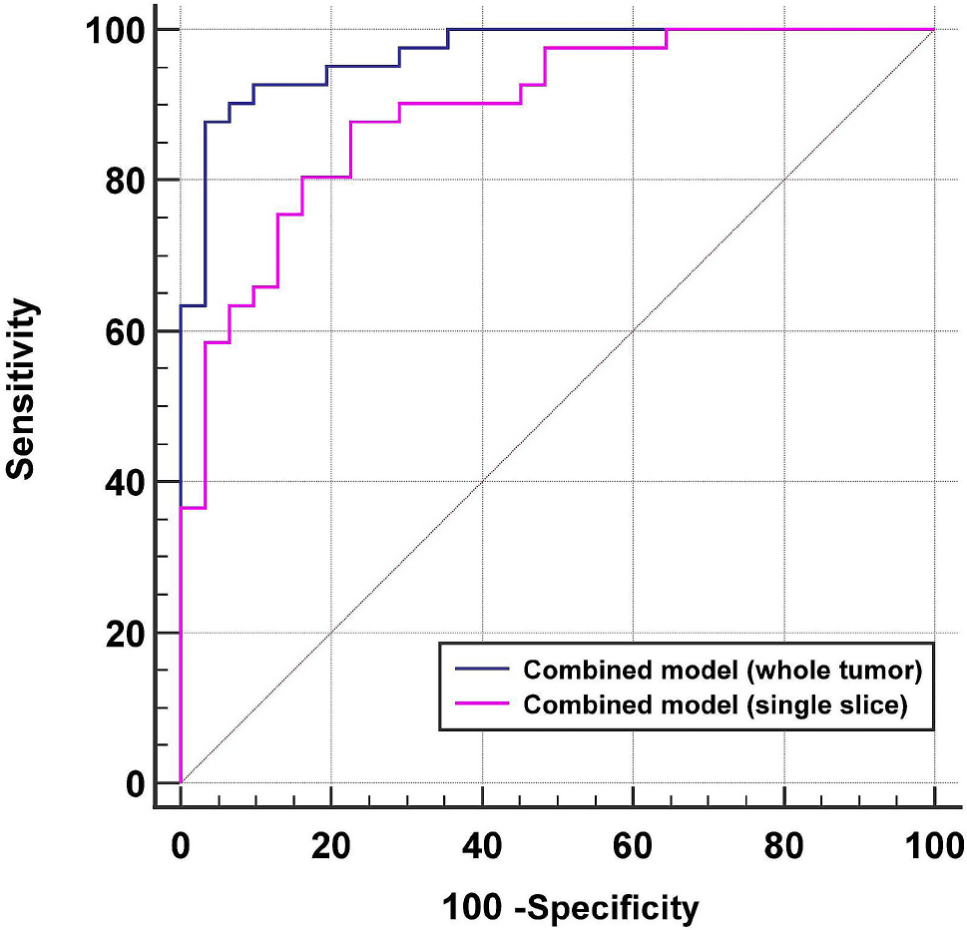
Receiver operating characteristic curves of the combined model obtained by single slice analysis method and whole tumor volume method for predicting treatment response to chemoimmunotherapy

The diagnostic performance of signifcant parameters and the combined model obtained by using whole tumor and single slice ROI analysis is summarized in Table 5; Fig. 3 and Supplementary Table 3, respectively. The mean value of f obtained from whole tumor and single slice both had the highest AUC (AUC = 0.886 and 0.812, respectively) compared to other parameters. The AUC of the mean f value from whole tumor volume was higher than the mean f value from single slice ROI (*P* = 0.044). Besides, for other variables, the AUC of the mean value was higher than or equivalent to that of other histogram metrics.

**Table 5:** Diagnostic performance of signifcant parameters and the combined model obtained by using whole tumor analysis

Parameters	AUC	Cutoff value	Youden Index	Sensitivity	Specificity	PPV	NPV	Accuracy	*P*
ADC									
75th	0.661	≤ 1.845	0.2730	85.37	41.94	66.04	68.43	66.65	0.0149
Mean	0.712	≤ 1.55	0.3611	78.05	58.06	71.11	66.67	69.43	0.0009
Median	0.649	≤ 1.39	0.3761	63.41	74.19	76.46	60.53	68.06	0.0296
Dk									
75th	0.640	≤ 1.65	0.3021	46.34	83.87	79.16	54.17	62.52	0.0315
90th	0.662	≤ 1.962	0.2880	70.73	58.06	69.04	60.00	65.27	0.0116
Mean	0.672	≤ 1.483	0.3525	70.73	64.52	72.50	62.50	68.05	0.0084
Dslow									
10th	0.730	≤ 0.817	0.4823	80.49	67.74	76.74	72.41	74.99	0.0002
25th	0.660	≤ 1.013	0.3446	73.17	61.29	71.42	63.34	68.05	0.0194
75th	0.759	≤ 1.599	0.5224	78.05	74.19	79.99	71.87	76.39	< 0.0001
90th	0.708	≤ 1.896	0.3611	78.05	58.06	71.11	66.67	69.43	0.0013
Mean	0.804	≤ 1.299	0.5869	78.05	80.65	84.21	73.54	79.17	< 0.0001
Median	0.779	≤ 1.28	0.6357	82.93	80.65	85.00	78.13	81.94	< 0.0001
f									
25th	0.747	> 7.834	0.4579	78.05	67.74	76.19	70.00	73.61	< 0.0001
75th	0.844	> 27.279	0.6593	75.61	90.32	91.17	73.69	81.95	< 0.0001
90th	0.793	> 40.801	0.5382	73.17	80.65	83.33	69.45	76.39	< 0.0001
Mean	0.886	> 17.958	0.6924	85.37	83.87	87.50	81.26	84.72	< 0.0001
Median	0.856	> 13.992	0.6522	87.80	77.42	83.72	82.76	83.33	< 0.0001
Combined model	0.968	> 0.693	0.8458	87.80	96.77	97.29	85.71	91.67	< 0.0001

AUC: area under the curve; PPV: positive predictive value; NPV: negative predic-tive value

After stepwise logistic regression analysis, the combined model of whole tumor volume was composed of three parameters (Dslow_mean_, f_mean_ and f_90th_) with an AUC of 0.968, which had significantly better diagnostic ability than optimal single parameter. Moreover, the combined model of single slice ROI was composed of another three parameters (Dslow_mean_, Dslow_75th_ and f_90th_) with an AUC of 0.893, which was statistically significantly higher than any single parameter except f_mean_ (AUC = 0.812, *P* = 0.0671), f_medium_ (AUC = 0.810, *P* = 0.0780), f_75th_ (AUC = 0.811, *P* = 0.0728) and f_90th_ (AUC = 0.806, *P* = 0.0513). The comibed model of whole tumor volume demonstrated a tendency toward higher AUC (0.968) than that of single slice ROI (0.893) for predicting treatment response, but this difference did not reach statistical significance (*P* = 0.0559). The results of stepwise logistic regression analysis are listed in Table 6.

**Table 6:** The results of stepwise logistic regression analysis

Combined model	β Coefficients	Standard error	Wald	OR (95%CI)	P	Model fit^a^
Whole tumor						0.789
Dslow_mean_	-0.567	0.238	5.678	0.567 (0.356–0.904)	0.017	
f_mean_	1.231	0.402	9.379	3.425 (1.558–7.530)	0.002	
f_90th_	-0.519	0.194	7.137	0.595 (0.407–0.871)	0.008	
Single slice						0.612
Dslow_mean_	-2.081	0.670	9.652	0.125 (0.034–0.464)	0.002	
Dslow_75th_	1.130	0.502	5.063	3.095 (1.157–8.280)	0.024	
f_90th_	0.087	0.027	10.564	1.091 (1.035–1.150)	0.001	

^a^The Hosmer–Lemeshow test was performed to explain the goodness-of-fit of the multivariate logistic model. A *P* > 0.05 was considered well fitted. OR: odds ratio; CI: confidence interval

Given that the diagnostic performance of the mean values of Dslow, f, Dk, and ADC was higher than that of other histogram parameters of these parameters, a model composed of Dslow_mean_, f_mean_, Dk_mean_, and ADC_mean_ was also established, the AUCs of this model were 0.935 (whole tumor analysis) and 0.858 (single slice analysis) respectively (Supplementary Table 4), which was lower than the AUC of the model established by stepwise logistic regression analysis.

## Discussion

This study found that the histogram parameters of ADC, Dk, Dslow and fmay be used to predict the therapeutic efficacy of immunochemotherapy in advanced NSCLC, and the diagnostic ability of the mean value of each parameter was better than or equivalent to other histogram metrics, where the mean value of f was suggested to be the most powerful predictive indicator. Moreover, the diagnostic efficiency of the combined model was better than that of optimal single parameter. For the two ROI selection methods, whole tumor volume analysis showed better diagnostic performance compared with single slice ROI analysis.

Previous studies have demonstrated that the pre-treatment ADC values derived from conventional DWI are imaging biomarkers to evaluate the treatment outcomes of various tumors [24–26], including lung cancers [27]. And the results of these studies shown that lower baseline ADC values implied better treatment efficacy, which was similar to our findings. Higher ADC values indicate more necrotic areas within the tumor, where tumor cells become less sensitive to chemotherapeutic drugs due to being in a hypoxic and acidic environment. And necrotic areas are usually poorly perfused, resulting in relatively fewer chemotherapy drugs being delivered to these areas [26]. In addition, tumors are more vulnerable to therapeutic drugs when in a proliferation state [25]. Ki67 index is an indicator reflecting the proliferation status of tumors, our study revealed that the proliferation index Ki67 was significantly higher in the responders groups, prior studies have confirmed a negative correlation between Ki67 index and ADC values in lung cancer [28–30] .

In this study, similar to ADC values, lower Dslow values before treatment also indicated more sensitive treatment response of immunochemotherapy, and the diagnostic performance of the Dslow value was superior to the ADC value, which was consistent with previous research results [31–33]. This result can be explained by the imaging principles of IVIM, which can effectively separate the diffusion of pure water molecules and the microcirculation components of tissues using the bi-exponential model, therefore, IVIM-derived Dslow value can more truly reflect the diffusion of water molecules. Correspondingly, the Dslow value is also significantly lower than the ADC value ascribed to the lack of the influence of the blood microcirculation in capillaries. Perucho et al. [34] and Zhang et al. [35] found high f value was associated with the sensitivity to concurrent chemoradiotherapy, our study results also show responders group had higher f value than non-responders group. f value measures the fractional blood volume in the capillary network, which reflects microscopic translational motion associated with microcirculation of the blood [12], the higher f value indicates higher proportion of microcirculation, richer blood supply, fewer hypoxic cells, more active proliferation and division, which can allow more drugs to be transported to tumor target [35]. However, our research results shown another perfusion-related parameter Dfast had limited value in the predition of treatment response, possibly due to the low stability and large standard deviation of Dfast [11] .

DKI-derived D_k_ parameter is the corrected diffusion coefficient accounting for non-Gaussian behavior, and K derived by DKI reflects the complexity of organizational microstructure. Some studies [24, 26, 36, 37] have confirmed that pre-treatment DKI parameters have good diagnostic performance for evaluating the efficacy of tumor treatment. In this study, the mean, 75th and 90th percentiles of Dk obtained from whole tumor and 90th percentile of Dk obtained from single slice could be used to predict chemoimmunotherapy response, but their diagnostic ability was not yet satisfactory with a maximum AUC of 0.672. None of the histogram parameters derived from K could be regarded as a promising tool for monitoring response to chemoimmunotherapy for patients with advanced NSCLC. The possible reasons for this result may be differences in tumor types and treatment strategies, as well as differences in ROI selection. In addition, the selection of models and the use of b-values can also have an impact. Our study selected three b values with the maximum b value being 2000 s/mm^2^. Technically, the highest b value using in DKI needs to reach > 1500 s/mm^2^ [11] .

Previous studies have mostly selected a representative section to outline ROI and obtain DWI parameters for evaluating the treatment response of lung cancer [9, 19, 27, 38]. Although this is a convenient and practical approach, the selection of ROI size and placement location may cause inaccuracies in the measurement results, and this method also ignores the heterogeneity of the entire tumor. Several studies have found that the whole tumor analysis method had higher repeatability in DWI parameter measurement compared to single slice ROI analysis method [21, 39]. In the present study, the histogram parameter measurement repeatability of the two ROI selection methods( whole tumor and single slice) was good to excellent. Furthermore, the whole tumor method could obtain more statistically significant parameters for predicting treatment response. The diagnostic performance of the whole tumor method was superior to that of the single slice method, because the analysis based on the whole lesion involves all components within the lesion, thus better reflecting the inherent intratumoral heterogeneity. Therefore, we should choose the whole volume method for the measurement of the parameters in order to be able to assess the lesion more accurately. Interestingly, our results indicated that the mean value obtained by the entire volume method had better diagnostic efficacy than other histogram parameters, as described previously [36]. Thus, we can use the mean value obtained from the whole tumor volume to assess the tumor response to chemoimmunotherapy in lung cancer, thereby avoiding more parameter measurements and analysis. Additionally, due to the complementarity of clinical values among various parameters, the combination model can significantly improve the diagnostic efficiency, which would have the potential to become an alternative diagnostic method.

This study has some limitations. First, this is a single center study and the sample size is not large. Second, due to respiratory motion artifacts causing poor image quality, some patients were excluded. We use breath gating and set appropriate acquisition windows to reduce motion interference. Third, the follow-up period is not long enough, future research needs to evaluate outcome and survival of NSCLC with chemoimmunotherapy. Fourth, there is no standard scanning parameters for the IVIM and DKI sequence. And there is no consensus on the quantity and size of b values either. Finally, volumetric analysis may include misregistration artifacts, therefore we did not record the extreme values of each parameter.

## Conclusions

Our preliminary results indicate the histogram parameters of ADC, IVIM and DKI hold the potential to predict the response to chemoimmunotherapy in advanced NSCLC. The mean value yields better diagnostic efficiency, which can avoid analyzing more histogram parameters in routine clinical practice, and the combined model improved the prediction performance. Whole tumor volume can better capture the intratumoral heterogeneity, and its diagnostic performance is superior to single slice analysis.

### Electronic supplementary material

Below is the link to the electronic supplementary material.

Supplementary Material 1

## Data Availability

The datasets used and/or analysed during the current study are available from the corresponding author on reasonable request.

## Abbreviations

NSCLC: Non-small cell lung cancer
DWI: Diffusion-weighted imaging
IVIM: Intravoxel incoherent motion
DKI: Diffusion kurtosis imaging
ROI: Region of interest
TR/TE: Repetition time/echo time
FOV: Field of view
ADC: Apparent diffusion coefficient
Dslow: True diffusion coefficient
Dfast: Pseudo-diffusion coefficient
f: Perfusion fraction
Dk: Corrected diffusion coefficient
K: Diffusion kurtosis value
RECIST: Response Evaluation Criteria in Solid Tumors
CR: Complete response
PR: Partial response
PD: Progressive disease
SD: Stable disease
ICC: Intraclass correlation coefficient
ROC: Receiver operating characteristic curve
AUC: Area under the curve
PPV: Positive prediction value
NPV: Negative prediction value
