# Supplementary material for: Histogram analysis of multiple diffusion models for predicting advanced non-small cell lung cancer response to chemoimmunotherapy
Source: Cancer Imaging. 2024 Jun 11;24:71. doi: 10.1186/s40644-024-00713-8 (PMC11167789; doi:10.1186/s40644-024-00713-8)
Supplement: Supplementary file 1 — Supplementary Material 1 [file 40644_2024_713_MOESM1_ESM.docx]

**Supplementary Table 1.** **Comparisons of ADC and DKI histogram metrics obtained** **by using single slice analysis**

| **Parameters** | **ADC** |  | **P** | **Dk** |  | **P** | **K** |  | **P** |
| --- | --- | --- | --- | --- | --- | --- | --- | --- | --- |
|  | **Responders**  **(n=41)** | **Non-responders**  **(n=31)** |  | **Responders**  **(n=41)** | **Non-responders**  **(n=31)** |  | **Responders**  **(n=41)** | **Non-responders**  **(n=31)** |  |
| 10th | 0.97 ± 0.16 | 0.98 ± 0.15 | 0.687 | 0.94 ± 0.16 | 0.96 ± 0.14 | 0.560 | 0.49 ± 0.22 | 0.46 ± 0.25 | 0.495 |
| 25th | 1.14 ± 0.18 | 1.19 ± 0.18 | 0.214 | 1.16 ± 0.18 | 1.20 ± 0.17 | 0.409 | 0.74 ± 0.23 | 0.68 ± 0.25 | 0.290 |
| 75th | 1.57 ± 0.27 | 1.73 ± 0.26 | 0.009 | 1.68 ± 0.21 | 1.77± 0.17 | 0.073 | 1.17 ± 0.26 | 1.11 ± 0.35 | 0.401 |
| 90th | 1.82 ± 0.31 | 1.96 ± 0.29 | 0.057 | 1.94 ± 0.25 | 2.07 ± 0.25 | 0.039 | 1.35 ± 0.27 | 1.33 ± 0.47 | 0.298 |
| Mean | 1.40 ± 0.19 | 1.57 ± 0.25 | 0.001 | 1.45 ± 0.22 | 1.54 ± 0.15 | 0.070 | 0.94 ± 0.22 | 0.89 ± 0.29 | 0.419 |
| Median | 1.35 ± 0.22 | 1.47 ± 0.25 | 0.002 | 1.41 ± 0.23 | 1.50 ± 0.17 | 0.072 | 0.98 ± 0.25 | 0.88 ± 0.29 | 0.136 |
| Skewness | 1.25 ± 1.03 | 2.00 ± 2.70 | 0.339 | 0.74 ± 1.25 | 0.83 ± 1.39 | 0.761 | -0.26 ± 0.71 | -0.07 ± 0.74 | 0.272 |
| kurtosis | 2.98 ± 6.05 | 2.94 ± 3.84 | 0.348 | 1.03 ± 2.17 | 1.53 ± 2.55 | 0.513 | 0.42 ± 1.20 | 0.85 ± 2.05 | 0.550 |
| Entropy | 2.58 ± 1.09 | 2.29 ± 0.91 | 0.243 | 3.50 ± 0.31 | 3.56 ± 0.29 | 0.433 | 3.85± 0.37 | 3.93 ± 0.27 | 0.629 |

**Supplementary Table 2. Comparisons of IVIM histogram metrics obtained by using single slice analysis**

| **Parameters** | **Dslow** |  | **P** | **Dfast** |  | **P** | **f** |  | **P** |
| --- | --- | --- | --- | --- | --- | --- | --- | --- | --- |
|  | **Responders**  **(n=41)** | **Non-responders**  **(n=31)** |  | **Responders**  **(n=41)** | **Non-responders**  **(n=31)** |  | **Responders**  **(n=41)** | **Non-responders**  **(n=31)** |  |
| 10th | 0.82 ± 0.12 | 0.91 ± 0.14 | 0.003 | 3.90 ± 2.52 | 3.06 ± 2.13 | 0.103 | 4.37 ± 2.99 | 3.28 ± 2.67 | 0.063 |
| 25th | 1.00 ± 0.15 | 1.11 ± 0.15 | 0.004 | 7.13 ± 4.05 | 5.64 ± 2.72 | 0.144 | 12.10 ± 6.76 | 7.50 ± 4.58 | 0.002 |
| 75th | 1.46 ± 0.22 | 1.62 ± 0.22 | 0.003 | 18.19 ± 8.34 | 17.00 ± 5.46 | 0.754 | 33.13 ± 13.41 | 20.00 ± 7.93 | 0.000 |
| 90th | 1.70± 0.26 | 1.80 ± 0.23 | 0.079 | 28.02 ± 11.00 | 26.63 ± 7.78 | 0.737 | 44.98 ± 15.62 | 28.77 ± 12.49 | 0.000 |
| Mean | 1.25 ± 0.16 | 1.44 ± 0.18 | 0.000 | 13.28 ± 5.32 | 12.63 ± 3.98 | 0.581 | 24.04 ± 10.22 | 14.56 ± 5.96 | 0.000 |
| Median | 1.21 ± 0.16 | 1.40 ± 0.20 | 0.000 | 11.54 ± 5.98 | 9.83 ± 3.44 | 0.334 | 22.80 ± 9.67 | 12.62 ± 4.80 | 0.000 |
| Skewness | 1.04 ± 0.96 | 1.11 ± 1.10 | 0.306 | 1.74 ± 1.29 | 1.51 ± 0.82 | 0.789 | 1.10 ± 1.02 | 1.34 ± 0.82 | 0.217 |
| kurtosis | 1.94 ± 2.16 | 3.04 ± 3.04 | 0.226 | 3.24 ± 4.76 | 2.12 ± 2.35 | 0.363 | 1.30 ± 2.18 | 2.15 ± 2.11 | 0.055 |
| Entropy | 3.57 ± 0.38 | 3.61 ± 0.43 | 0.613 | 1.27 ± 0.78 | 1.36 ± 0.60 | 0.211 | 3.81 ± 0.66 | 3.77 ± 0.28 | 0.784 |

**Supplementary Table 3. Diagnostic performance of signifcant parameters and the combined model obtained by using single slice analysis**

| **Parameters** | **AUC** | **Cutoff**  **value** | **Youden**  **Index** | **Sensitivity** | **Specificity** | **PPV** | **NPV** | **Accuracy** | **P** |
| --- | --- | --- | --- | --- | --- | --- | --- | --- | --- |
| ADC |  |  |  |  |  |  |  |  |  |
| 75th | 0.626 | ≤1.879 | 0.2651 | 87.80 | 38.71 | 65.45 | 70.58 | 66.64 | 0.0652 |
| Mean | 0.663 | ≤1.640 | 0.2895 | 90.24 | 38.71 | 66.07 | 75.00 | 68.03 | 0.0124 |
| Median | 0.668 | ≤1.634 | 0.2573 | 90.24 | 35.48 | 64.91 | 73.33 | 66.64 | 0.0296 |
| Dk |  |  |  |  |  |  |  |  |  |
| 90th | 0.609 | ≤1.845 | 0.2368 | 36.59 | 87.10 | 78.95 | 50.95 | 58.36 | 0.1028 |
| Dslow |  |  |  |  |  |  |  |  |  |
| 10th | 0.649 | ≤0.886 | 0.3202 | 70.73 | 61.29 | 70.73 | 61.29 | 66.66 | 0.0235 |
| 25th | 0.649 | ≤0.973 | 0.3265 | 48.78 | 83.87 | 80.00 | 55.32 | 63.90 | 0.0230 |
| 75th | 0.673 | ≤1.454 | 0.3761 | 63.41 | 74.19 | 76.46 | 60.53 | 68.06 | 0.0095 |
| Mean | 0.745 | ≤1.301 | 0.4815 | 70.73 | 77.42 | 80.55 | 66.67 | 73.61 | 0.0001 |
| Median | 0.719 | ≤1.292 | 0.4571 | 68.29 | 77.42 | 80.00 | 64.87 | 72.23 | 0.0007 |
| f |  |  |  |  |  |  |  |  |  |
| 25th | 0.724 | >10.167 | 0.3674 | 56.10 | 80.65 | 79.31 | 58.15 | 66.68 | 0.0002 |
| 75th | 0.811 | >22.549 | 0.4902 | 78.05 | 70.97 | 78.05 | 70.97 | 75.00 | <0.0001 |
| 90th | 0.806 | >27.187 | 0.4988 | 85.37 | 64.52 | 76.09 | 76.93 | 76.38 | <0.0001 |
| Mean | 0.812 | >19.666 | 0.4729 | 63.41 | 83.87 | 83.87 | 56.94 | 72.23 | <0.0001 |
| Median | 0.810 | >18.879 | 0.5374 | 63.41 | 90.32 | 89.65 | 65.12 | 75.01 | <0.0001 |
| Combined model | 0.893 | >0.410 | 0.6522 | 87.80 | 77.42 | 83.72 | 82.76 | 83.33 | <0.0001 |

AUC: area under the curve; PPV: positive predictive value; NPV: negative predic-tive value.

**Supplementary Table 4. Diagnostic performance of the model composed of Dslow_mean_, f_mean_, Dk_mean_, and ADC_mean_**

| **Parameters** | **AUC** | **Cutoff**  **value** | **Youden**  **Index** | **Sensitivity** | **Specificity** | **PPV** |  | **NPV** | **Accuracy** | **P** |
| --- | --- | --- | --- | --- | --- | --- | --- | --- | --- | --- |
| Whole tumor | 0.935 | >0.459 | 0.798 | 92.68 | 87.10 | 90.48 |  | 90.00 | 90.28 | <0.0001 |
| Single slice | 0.858 | >0.644 | 0.578 | 70.73 | 87.10 | 87.88 |  | 69.23 | 77.79 | <0.0001 |
